# Do CEOs with government work experience foster enterprise investment in pollution control?

**DOI:** 10.1371/journal.pone.0317903

**Published:** 2025-01-24

**Authors:** Mixiang Peng, Dayuan Li, Chaolin Zhang

**Affiliations:** 1 School of Business Administration, Zhejiang Gongshang University, Hangzhou, PR China; 2 School of Business, Central South University, Changsha, PR China; 3 The School of Finance, Hunan University of Technology and Business, Changsha, PR China; Durham University, UNITED KINGDOM OF GREAT BRITAIN AND NORTHERN IRELAND

## Abstract

As enterprise leaders, CEOs play a critical role in driving enterprise investment in pollution control. However, few studies have explored the motivations behind enterprise investment in pollution control, primarily how CEOs’ early experiences influence their decisions. Based on the perspective of imprinting theory, this study examines the impact of CEOs with government work experience on enterprise investment in pollution control and the boundary conditions of this impact. Using data from a survey of private enterprises in China from 2008 to 2014, the empirical results indicate that CEOs with government work experience are likelier to promote enterprise investment in pollution control. Additionally, a CEO’s perceptions of economic, social, and political status negatively moderate the relationship between their government work experience and enterprise investment in pollution control. This study enriches and deepens the study on career imprinting in non-commercial fields, status perception, and sustainable development and provides practical significance for hiring CEOs with government work experience to promote enterprise investment in pollution control.

## 1. Introduction

As leaders of enterprises, CEOs bear significant responsibility for environmental governance, particularly concerning pollution control investments [1, 2]. They directly influence an enterprise’s environmental performance by making strategic decisions, allocating resources, and ensuring the effective implementation of environmental measures [3]. For example, Andrew Liveris, the former CEO of Dow Chemical, leveraged his experience in developing environmental policies within international organizations to drive significant investments in pollution control technologies. However, CEOs exhibit varying performances in pollution control investments, indicating that a CEO’s background may significantly impact this disparity [4]. For instance, former Volkswagen CEO Martin Winterkorn’s experiences in the traditional automotive industry led him to prioritize profits over environmental responsibility, ultimately resulting in the emissions cheating scandal. Therefore, investigating how CEO’s past experiences influence enterprises’ investment in pollution control can provide valuable insights for leadership selection and enhance our understanding of enterprise environmental responsibility mechanisms.

In recent years, academia has conducted detailed research on the relationship between CEOs and enterprise environmental governance, which can be categorized into two types. The first type focuses on the innate traits of CEOs, such as gender [5], age [6], overconfidence [7], and emotional intelligence [8]. The second type examines how CEOs’ acquired experiences influence enterprise environmental behavior. Existing studies indicate that CEOs with delegated authority [9], marketing experience [2], and professional backgrounds [10] significantly influence environmental governance. Unlike innate traits, acquired experiences provide CEOs with specialized knowledge and skills and shape their decision-making styles and cognitive patterns [11]. Consequently, scholars have explored the influence of acquired experiences on environmental governance, examining it from the perspectives of both commercial and non-commercial experiences.

Existing research has found a significant association between CEOs commercial experiences—such as marketing experience [2] and financial experience [12]—and environmental governance. CEOs’ commercial experiences are typically aligned with enterprise economic development and contribute to improving enterprise performance [13]. By implementing environmental governance, CEOs aim to gain social recognition for their enterprises, enhance enterprise image, and promote economic growth. Therefore, environmentally driven behaviors influenced by commercial experience primarily aim to serve enterprise interests and are driven by external benefits [12, 14].

By contrast, CEOs with non-commercial experiences tend to exhibit environmental governance behaviors driven more by an internal sense of responsibility after entering profit-oriented enterprises. They are more likely to focus on fulfilling social responsibilities rather than pursuing economic benefits [11, 15]. This shift may lead them to face conflicts and dilemmas between economic development and social responsibility in their decision-making [16]. Against this backdrop, scholars have gradually begun to explore the impact of CEOs’ non-commercial experiences on enterprise environmental governance. For instance, Zhang et al. [15] note that CEOs and board chairs with military backgrounds are more inclined to adopt environmentally friendly decisions when facing conflicts between economic development and social responsibility. Thus, while promoting enterprise economic growth, they focus on environmental governance, driving pollution control, and environmental innovation, which helps mitigate this conflict.

In addition to military experience, government work experience is another critical form of non-commercial experience for a CEO. However, research on the relationship between CEOs with government work experience and enterprise environmental governance remains incomplete [4], particularly regarding the lack of studies focusing on the connection between CEOs with government work experience and enterprise investment in pollution control. Despite the scarcity of related research, the service experience of CEOs in government departments can significantly enhance their attention to environmental governance and sustainable development issues [17, 18], as well as their relevant knowledge and capabilities. This experience may influence their decision-making processes within enterprises and directly drive enterprise investment in pollution control. Therefore, a deeper exploration of the relationship between CEOs with government work experience and enterprise investment in pollution control, along with its potential mechanisms, is critical for research.

Imprinting theory offers a compelling perspective on this issue. The imprinting theory posits that the characteristics formed during an individual’s sensitive periods continue influencing their behavior and decision-making patterns even in dynamic environments [19, 20]. Simsek [19] indicates that the beginning of an individual’s career is an important and sensitive period because it represents a critical transition from education to the workforce. Even if they later change careers, their experiences during this early sensitive period will continue to impact their cognitive decision-making [21, 22]. When individuals leave government positions to work in the enterprise sector, they continue exhibiting decision-making behaviors characteristic of their government work, a phenomenon known as government imprinting. Therefore, government imprinting may be why CEOs with government work experience can promote enterprise investment in pollution control.

Furthermore, Simsek [19] suggests that imprints undergo dynamic changes after formation, including persisting, amplifying, decay, or transformation. When an individual leaves a government position and becomes a CEO, their status perception changes, affecting the impact of the government imprinting. Status perception refers to an individual’s sense of value and respect based on their relative position within a specific group [23]. Weber [24] categorizes status perception into three dimensions: economic, social, and political, providing a practical framework for exploring how different types of status perception influence CEO decision-making. Research indicates that various dimensions of status perception can affect CEOs’ decision-making styles. For instance, CEOs with high economic status perception are more likely to focus on short-term financial performance [25], while those with high social status perception prioritize enterprise social reputation [26]. In the context of enterprise investment in pollution control, a CEO’s status perception may affect their environmental governance decisions by altering their responses to external pressures. Therefore, this study employs Weber’s three-dimensional status perception framework to examine how different status perceptions affect the decision-making tendencies of CEOs with government work experience.

Based on this, our study integrates imprinting theory and status perception concepts to explore two essential but under-researched questions: How does a CEO with government work experience influence enterprise investment in pollution control? Second, does status perception strengthen or weaken the impact of a CEO with government work experience on enterprise investment in pollution control?

This study utilized survey data from Chinese private enterprises from 2008 to 2014 to investigate the relationship between CEOs with government work experience and enterprise investment in pollution control and the moderating role of status perception. The choice of Chinese private enterprises as the research sample was due to their significant role in China’s economic development and environmental protection as representative of a developing country [27, 28]. The research findings indicate that CEOs with government work experience facilitate enterprise investment in pollution control. Furthermore, the moderation analysis reveals that perceptions of economic, social, and political statuses negatively moderate the positive relationship between CEOs with government work experience and enterprise investment in pollution control.

This study contributes to the literature in three main aspects. First, it examines the impact of CEOs with government work experience on enterprise investment in pollution control. This enriches the understanding of non-commercial experience career imprints and expands the application of imprinting theory in environmental governance [4, 11, 20]. We define the decision-making behavior formed by individual government work experience as “government imprinting,” providing a new theoretical perspective for non-commercial experience career imprints. Second, we delineate the dimensions of status perception and analyze how these dimensions influence the relationship between CEOs with government work experience and enterprise investment in pollution control. This enriches the conceptual connotation and practical application of status perception [29, 30]. It contributes to the relevant literature, offering a theoretical basis for enterprises to utilize status perception in different contexts. Finally, this study analyzes the impact of CEOs with government work experience on enterprise investment in pollution control from a micro-level perspective, emphasizing the role of individual government work experience and intrinsic sense of responsibility in promoting sustainable development. This study not only responds to the call by Mahran and Elamer [4] regarding how CEO experience affects environmental sustainability practices and outcomes but also offers new insights into the field of sustainable development.

## 2. Theoretical background and hypothesis development

### 2.1 Imprinting theory and government imprinting

Initially proposed by Stinchcombe [31], the imprinting theory suggests that the characteristics imprinted on organizations and individuals during sensitive periods persistently influence their behavior and decision-making patterns, even in dynamic environments [32]. Specifically, the theory entails three aspects: (1) the sensitive period, during which organizations and individuals are susceptible to external influences while undergoing transition; (2) the imprinting process, whereby organizations and individuals subconsciously adopt the behavior and decision-making patterns imparted by the environment during interaction; and (3) the persistent effect of imprints despite subsequent changes in the environment [19, 20].

Marquis and Tilcsik [20] indicate that early career experiences significantly impact individuals’ career trajectories. These early experiences promote enterprise development, as observed in widely studied areas such as financial experience [33], entrepreneurial experience [34], and marketing experience [2]. However, research on the impact of non-commercial experiences, such as military experience [15], on enterprise development is relatively limited. Current studies primarily focus on the effects of CEOs’ commercial experiences on enterprise development, while exploration of non-commercial experiences, particularly government work experience, must be improved. CEOs with government work experience can provide unique advantages for enterprise development, such as enhanced policy understanding and influence, as well as risk management and coping abilities [35, 36]. Therefore, this area warrants further attention.

Individuals working in government departments are referred to as civil servants. Civil servants are the executors of government policies and constitute the leading force in public administration and social governance [36, 37]. They are widely distributed across party and government organs, government departments, public institutions, and grassroots mass autonomous organizations. When civil servants leave government departments and enter the enterprise sector, they exhibit decision-making behavior characterized by governmental work, known as government imprinting.

Government imprinting is a multistage process primarily shaping individuals’ behavior and decision-making through structure, cognition, and culture [19]. First, during individuals’ sensitive periods, there is a restructuring of roles at the structural level. Approximately 70% of talents recruited by government departments are university graduates, a phase coinciding with the early stages of their careers, also known as the sensitive period in imprinting theory [19, 20]. Individuals are more likely to accept and adapt to new work patterns and embrace government departments’ missions and responsibilities [38], thus undergoing structural role restructuring. Second, cognitive reshaping is used during the imprinting process. Governments at all levels select talents through rigorous and standardized procedures such as written tests, political reviews, and interviews [39, 40]. Precisely, written tests assess individuals’ stress-coping abilities and basic civil service knowledge, while political reviews examine their ideological qualities and interpersonal attitudes [41]. Interviews assess individuals’ comprehensive qualities, including knowledge, abilities, and ethics [40]. Through the complete selection process, individuals undergo cognitive reshaping. Third, after imprint formation, there is continuous cultural influence. Government departments regularly conduct training activities such as education on party discipline and history and specialized training to enhance capabilities [42, 43]. These activities convey the concept of serving the people and building a service-oriented government to individuals in diverse forms, thus maintaining continuous cultural influence.

Simsek et al. [19] further suggest that it undergoes dynamic changes after imprint formation, including imprints persist, amplify, decay, and transform. Civil servants are highly esteemed by the public due to their rigorous selection and professional training, responsibility for managing public resources, direct service to the public, and stable employment with favorable welfare benefits [44]. When individuals leave government departments and become CEOs of enterprises, their perception of status changes, affecting the effectiveness of government imprinting. Therefore, leveraging imprinting theory, we examine the impact of CEOs with governmental work experience on enterprise investment in pollution control and explore the moderating role of status perception.

### 2.2 CEOs’ government work experience and enterprise investment in pollution control

The imprinting theory posits that traits developed during an individual’s sensitive period have a lasting influence on their future behavior and decision-making [19, 20]. Government imprinting, a specific application of imprinting theory, refers to how experiences in government shape individuals’ subsequent decision-making patterns. CEOs with government work experience tend to exhibit unique behavior in enterprise investment in pollution control, which can be categorized into three aspects:

First, from a structural perspective, government work experience provides CEOs with a comprehensive framework for public management, particularly in risk management and crisis response. CEOs who possess government imprinting accumulate significant experience in managing environmental risks and public crises during their tenure in government [45, 46]. This background is instrumental when they transition into enterprise leadership roles, enabling them to recognize and address the long-term risks associated with environmental pollution, including legal, reputational, and financial vulnerabilities [47, 48]. Consequently, at the structural level, government imprinting encourages these CEOs to prioritize increased enterprise investment in pollution control initiatives to mitigate potential external risks.

Second, from a cognitive perspective, the mental frameworks developed through government work experience provide CEOs with distinct advantages in comprehending and responding to environmental policies and regulations [49]. During their early careers, the rigorous policy formulation and implementation processes within governmental contexts profoundly shaped their sensitivity to and foresight regarding environmental regulations. This heightened policy awareness enables them to anticipate regulatory changes effectively [4], facilitating the proactive adoption of measures that drive increased environmental investment within their organizations [3, 17]. Furthermore, their governmental background enhances their ability to communicate and collaborate effectively with government agencies [50, 51], thereby securing additional policy support and resources that further promote enterprise investment in pollution control.

Third, from a cultural perspective, the ingrained sense of responsibility and mission cultivated in the government work environment significantly influences CEOs’ behavior. Their extensive public service includes rigorous training in party discipline and social responsibility [42, 43], fostering a profound sense of social obligation [52]. This responsibility compels them to focus not only on their organizations economic interests but also to perceive the enterprise as part of society, willingly embracing greater social responsibilities [53, 54]. Consequently, these CEOs are inclined to advocate for increased enterprise investment in pollution control, fulfilling their commitments to environmental stewardship [55, 56] and enhancing their organizations’ social reputation.

In summary, CEOs with government work experience tend to drive enterprise investment in pollution control more vigorously due to the multifaceted influence of government imprinting across structural, cognitive, and cultural dimensions. These imprints enhance proactivity in addressing environmental risks, comprehending policy mandates, and assuming social responsibilities, thereby facilitating increased enterprise investments in pollution control efforts. Based on this, the study proposes the following hypothesis:

**Hypothesis 1.** CEOs with government work experience are positively associated with enterprise investment in pollution control.

### 2.3 Moderating effect of CEOs’ status perception

Early experiences working in the government impact CEOs’ cognition and decision-making, fostering investments in environmental governance within their enterprises. Although imprinting has a lasting effect on individuals, this effect is dynamic and adjusts with environmental changes [1, 19]. In China, organizational types are classified into five categories: government, state-owned enterprises, foreign enterprises, private enterprises, and others [27]. Government and state-owned enterprises are perceived as high-status organizations [27]. Therefore, when a CEO transitions from a government department to an enterprise, the government imprinting formed during the critical period still exists. However, changes in the work environment may alter their self-perceived status, thereby influencing the extent of the government imprinting effect.

Status perception refers to an individual’s sense of being valued and respected based on their relative status within a specific group [23]. Weber posited that individuals primarily assess their status based on their economic status (wealth and income), political status (power), and social status (prestige) [24]. Previous research has demonstrated that a CEO’s economic, political, and social status can influence decision-making in different enterprise aspects [27, 28, 38]. Therefore, we will discuss in detail how the three dimensions of status perception each moderate the relationship between CEOs with government work experience and enterprise investment in pollution control.

#### 2.3.1 Moderating effect of CEOs’ economic status perception

When a CEO transitions from a governmental department to an enterprise, there are corresponding changes and fluctuations in their perceived economic status. This is due to significant differences in how salaries are distributed between governmental departments and enterprises. Salaries in governmental departments are relatively lower but stable [57], thus resulting in less fluctuation in the economic status perception. However, salaries are primarily based on individual contributions to enterprise performance [58, 59], leading to more significant variability and instability. Therefore, a CEO’s economic status perception becomes more volatile upon entering an enterprise. According to the imprinting theory, once individual imprinting is formed, dynamic changes in the surrounding environment also affect the effectiveness of this imprint [19, 20]. Based on this, a CEO’s economic status perception weakens the influence of the government imprinting, negatively moderating the positive correlation between a CEO’s governmental work experience and enterprise investment in pollution control. Specifically, this is manifested in the following two aspects:

First, the higher the CEO’s economic status perception, the more they become aware of the intensity of the enterprise’s competitive environment, thus weakening the influence of their government imprinting cognitively. When a CEO has a higher economic status perception, they gain a deeper understanding of the enterprise’s internal and external competitive dynamics [60]. Enterprises must continually innovate and enhance efficiency to maintain and improve their competitive edge [61]. This intense competition demands CEOs to direct more attention and resources to projects that enhance enterprise performance, such as market expansion, product development, and cost control [62, 63]. Conversely, pollution control investment, while essential, may have lower short-term economic returns and could therefore be deprioritized [64]. Consequently, owing to the pressure of this competitive environment, CEOs gradually diminish the impact of their early governmental work experience on their awareness of pollution control [65], shifting their focus toward the enterprise’s economic performance and market competitiveness.

Second, the higher the CEO’s economic status perception, the more they can perceive the differences between the development of enterprises and governmental departments, thus weakening the influence of their government imprinting structurally. CEOs with a high economic status perception can better understand the fundamental disparities between enterprise and governmental operations [18]. Governmental departments prioritize public service and social responsibility [18], with decision-making processes being relatively stable and standardized and supported by national financial backing, resulting in stable salaries and positions. In contrast, enterprises primarily aim for profitability, with decision-making being flexible and responsive to market changes, salaries, and positions closely tied to individual performance [4, 64]. These operational differences lead high-economic-status-perceiving CEOs to prioritize flexibility and market orientation in enterprise management rather than the stability and standardization of governmental departments [66, 67]. As they gain a deeper understanding of enterprise structure and development models, the pollution control awareness and sense of social responsibility formed during their early governmental experience gradually diminish in practical enterprise operations [52], weakening the influence of the government imprinting structurally.

High economic status perception makes CEOs more inclined toward flexible and market-oriented management approaches in cognition and structure [64], weakening the effect of the government imprinting. Consequently, CEOs with higher economic status perception focus more on the enterprise’s competitive environment and profit objectives [34, 68], diminishing the impact of their governmental work experience on pollution control investment. Based on this, the following hypothesis is proposed:

**Hypothesis 2a.** CEO economic status perception weakens the positive relationship between CEOs with government work experience and enterprise investment in pollution control.

#### 2.3.2 Moderating effect of CEOs’ social status perception

When CEOs transition from governmental departments to enterprises, their social status perception also changes. Governmental departments are characterized by public service and altruism [18], leading individuals working in such departments to be perceived as having higher social status by the public [27]. Conversely, enterprises primarily aim for profit, offering goods and services for compensation [69]. CEOs’ social status perception influences the public’s evaluation of the enterprise’s products and services [70]. According to imprinting theory, dynamic changes in the surrounding environment can influence the sustained effects of imprinting [19, 20]. Therefore, CEOs’ social status perception weakens the impact of government imprinting, negatively moderating the positive correlation between CEOs with governmental work experience and enterprise investment in pollution control. Specifically, this is mainly manifested in the following two aspects:

First, the higher the CEO’s social status perception, the more likely they are to choose social responsibility actions that balance public interest with enterprise benefits [71], thereby weakening the influence of government imprinting on a cognitive level. The sources and manifestations of high social status perception are diverse [72, 73], and CEOs do not necessarily fulfill public expectations through enterprise investment in pollution control. They can achieve social responsibility goals through immediate measures such as charitable donations or by enhancing employee welfare [74, 75]. These measures can satisfy social expectations while promoting enterprise development, thus increasing employee motivation and improving enterprise performance. By contrast, enterprise investment in pollution control requires substantial ongoing financial resources and occupies economic capital with a more extended return period [76]. Therefore, on a cognitive level, CEOs with high social status perception may be more inclined to choose actions that can achieve social value in the short term, leading to a behavior orientation that weakens the impact of government imprinting when addressing environmental issues.

Second, the higher the CEO’s social status perception, the more influenced they are by the social networks of peer entrepreneurs, thereby culturally weakening the role of government imprinting. CEOs with a high perception of social status typically have broader social networks, particularly with similar entrepreneurs [77, 78]. These network relationships indicate that they are influenced by other entrepreneurs in their industry when making business decisions [79], leading them to focus more on operational efficiency than social responsibility. The entrepreneurial culture emphasizes business success and performance, relegating social responsibility to a secondary position. Frequent interactions with other entrepreneurs may prompt CEOs to pursue short-term economic benefits while neglecting long-term environmental responsibilities [80]. Therefore, on a cultural level, CEOs with high social status perceptions are influenced by the values of the entrepreneurial community [26], further diminishing the impact of government imprinting on pollution control decisions.

In summary, a high perception of social status among CEOs affects the effectiveness of government imprinting through cognitive and cultural dimensions, thereby weakening the positive relationship between CEOs with government work experience and enterprise investment in pollution control. Based on this, we propose the following hypothesis:

**Hypothesis 2b.** CEO social status perception weakens the positive relationship between CEOs with government work experience and enterprise investment in pollution control.

#### 2.3.3 Moderating effect of CEOs’ political status perception

When a CEO transitions from a governmental position to an enterprise role, their political status perception changes significantly. Government officials typically enjoy higher political status and power, often being directly influenced and supported by policies and the political environment in decision-making processes [37]. However, upon entering the enterprise sector, the CEO’s political status perception becomes more complex and variable as enterprise operations are primarily driven by market forces, requiring quick adaptation and response to market changes [22, 81]. Consequently, the CEO’s political status perception becomes more dependent on the enterprise’s performance in the market and its contribution to enterprise achievements [47, 82]. According to imprinting theory, changes in the individual’s environment can significantly moderate the effect of imprinting over time [19, 20]. Therefore, the CEO’s political status perception weakens the impact of government imprinting, negatively moderating the positive correlation between the CEO with governmental work experience and enterprise investment in pollution control. Specifically, this is manifested in the following two aspects:

First, the higher the CEO’s political status perception, the more likely they are to gain early access to dynamic national policy information, thereby weakening the influence of government imprinting on resource allocation. CEOs with a heightened political status perception can promptly acquire insights into policy changes and trends, enabling them to swiftly mitigate risks and adjust strategies [83]. However, because pollution control investment does not typically yield immediate economic returns or competitive advantages in the short term [84], these CEOs are more inclined to allocate resources toward projects such as innovation and market expansion, which can rapidly enhance enterprise performance [81]. Consequently, a heightened political status perception tends to lower the priority of enterprise investment in pollution control [15], diminishing the impact of government imprinting on pollution control investment decisions.

Second, the higher the CEO’s political status perception, the more they can leverage their political resources and connections to secure additional development opportunities for the enterprise, thereby weakening the influence of their government imprinting on strategic decisions. CEOs with a high political status perception typically possess extensive political resources and networks [85], enabling them to leverage these assets to secure policy incentives, government contracts, or other benefits for the enterprise [86]. In such cases, CEOs prioritize allocating resources and efforts toward projects that yield immediate economic benefits, such as entering new markets or upgrading technologies [87], rather than toward pollution control initiatives that may yield little tangible results in the short term. Although they may have developed a strong awareness of pollution control during their tenure in government [88], the urgent economic demands of the enterprise tend to diminish their focus on pollution control in practical enterprise management [64], thus weakening the influence of their government imprinting on strategic decisions.

CEOs with a high political status perception are more inclined to allocate resources toward projects that can rapidly enhance enterprise performance and utilize their political resources to secure more development opportunities [49, 89], thereby diminishing the influence of their government imprinting on pollution control decisions. Based on this, we propose the following hypothesis:

**Hypothesis 2c.** CEO political status perception weakens the positive relationship between CEOs with government work experience and enterprise investment in pollution control.

## 3. Research methodology

### 3.1 Sample and data source

China is an ideal empirical context for this study, given the crucial role private enterprises play in driving economic development and environmental protection [29]. Our research sample is obtained from the Chinese Private Enterprise Survey (CPES) database, updated biennially and widely used in organizational and management research on Chinese small and medium enterprises (SMEs). This database is recognized as an authoritative and reliable source for obtaining data on private enterprises in China [27, 28], providing the foundation for testing our hypotheses [90].

We select data from 2008, 2010, 2012, and 2014 to investigate the relationship between CEOs with government work experience and enterprise investment in pollution control for two reasons. First, the dataset provides unique, detailed information on CEOs’ government work experience and subjective status perception, which are critical variables for our study and not readily available in other data sources [91]. Second, the dataset offers comprehensive, high-quality information on private enterprises and their CEOs, ensuring that our sample accurately represents China’s private sector, thereby enhancing the robustness of our findings.

In recent years, numerous scholars have also leveraged CPES data from 2008 to 2014 to explore enterprise environmental issues. For instance, Fu et al. [92] use the 2012 data to explore the effects of environmental regulation on enterprise performance, while Yan and Xu [93] examine the impact of political party cooperation on enterprise environmental investment using data from 2006 to 2014.

### 3.2 Regression models

To test these hypotheses, we construct the following verification model: Model (1) tests the relationship between CEOs with government work experience and enterprise investment in pollution control. Models 2 to 4 mainly test the moderating effect of CEO status perception.

$$Logit(EI∙Pollution∙Control)=\ln\frac{EI∙Pollution∙Control}{1-EI∙Pollution∙Control}=\alpha_{0}+\alpha_{1}∙GWCEO+\alpha_{2}∙{CV}_{S}+\alpha_{3}∙Year+{\;\alpha }_{4}∙Area+\varepsilon$$ (1)

$$Logit(EI∙Pollution∙Control)=\ln\frac{EI∙Pollution∙Control}{1-EI∙Pollution∙Control}=\beta_{10}+\beta_{11}∙GWCEO+\beta_{12}∙ES∙Perception{+\beta_{13}∙ES∙Perception∙GWCEO+\beta_{14}∙CV}_{S}+\beta_{15}∙Year+\beta_{16}∙Area+\delta_{1}$$ (2)

$$Logit(EI∙Pollution∙Control)=\ln\frac{EI∙Pollution∙Control}{1-EI∙Pollution∙Control}=\beta_{20}+\beta_{21}∙GWCEO+\beta_{22}∙SS∙Perception{+\beta_{23}∙SS∙Perception∙GWCEO+\beta_{24}∙CV}_{S}+\beta_{25}∙Year+\beta_{26}∙Area+\delta_{2}$$ (3)

$$Logit(EI∙Pollution∙Control)=\ln\frac{EI∙Pollution∙Control}{1-EI∙Pollution∙Control}=\beta_{30}+\beta_{31}∙GWCEO{+\beta }_{32}∙PS∙Perception{+\beta_{33}∙PS∙Perception∙GWCEO+\beta_{34}∙CV}_{S}+\beta_{35}∙Year+\beta_{36}∙Area+\delta_{3}$$ (4)

Where GWCEO is the independent variable, which is the CEO with government work experience; ES perception, SS perception, and PS perception are the moderating variables, which are CEO status perception; and CVs are the control variables, specifically, CEO sex, CEO age, CEO political connection, enterprise age, enterprise industry, and provincial GDP. In addition, the year of the sample and enterprise area are considered control variables.

### 3.3 Variables and measures

#### 3.3.1 Dependent variable

To measure *enterprise investment in pollution control (EI pollution control)*, we also use a dummy variable that takes the value of 1 if the enterprise invested in pollution control and 0 otherwise. The final value range is 0–1, with an average value of 0.355. Our dataset shows that 4503 enterprises have invested in pollution control.

#### 3.3.2 Independent variable

Deputies to the National People’s Congress and Chinese People’s Political Consultative Conference members are the most important official contacts between Chinese private entrepreneurs and the Chinese government [89, 94]. Holding a post in the administrative department of the government does not depend on political connections because it is illegal for government officials to engage in private enterprise in China [94]. In the survey, we define CEOs with government work experience (GWCEO) by including a dummy variable that takes the value of 1 if the CEO has previously worked in the Party, government, or public institution and 0 otherwise.

#### 3.3.3 Moderating variables

In the survey, CEOs were asked to use the MacArthur Ladder Scale to assess their status from economic, social, and political dimensions [95], following Weber’s theory of social stratification [24, 26]. The initial question in the questionnaire was: “Compared to other members of society around you, where do you think you stand on the social ladder in terms of economic (social or political) aspects?” (Scores range from 1–10, with higher scores indicating weaker perceived status). We adopted the method proposed by Uddin [96] to classify CEOs’ perceived status into high and low groups to study their different impacts. Following the study by Li et al. [67], the respondents’ scores were reverse-coded for a more straightforward analysis. Specifically, we assigned a value of 1 to scores below 5, indicating high perceived status for the CEO, and a value of 0 to scores above 5, indicating low perceived status for the CEO.

#### 3.3.4 Control variables

We select several variables to control for probable influences on the independent variables. At the CEO level, we control for *CEO sex* by including a dummy variable that takes the value of 1 if the CEO is female and 0 if the CEO is male; *CEO age* is measured by the year of the questionnaire minus the CEO’s year of birth, and *CEO political connection* by including a dummy variable that takes the value of 1 if the CEO is a representative of the National People’s Congress or the Chinese People’s Political Consultative Conference, and 0 otherwise.

At the enterprise level, we measure the *enterprise age* as the year of the questionnaire minus the year the enterprise was registered as a private enterprise. According to the guidelines for environmental information disclosure of listed enterprises (Exposure Draft) in 2010 and combined with the characteristics of private enterprises, the five industries of mining, manufacturing, electricity, gas and water, construction, and transportation are considered heavy pollution industries, and the rest are light pollution industries. Therefore, we control the *enterprise industry* by including a dummy variable that takes the value of 1 if the enterprise is a heavy pollution industry and 0 otherwise. We measure *provincial GDP* using the natural logarithm of the previous year’s provincial GDP. To account for whether area conditions play a role in enterprise investment in pollution control, we control the *enterprise area* using a dummy variable that takes the value of 1 if the enterprise belongs to the eastern area and 0 otherwise. Finally, we control for time effects by including the corresponding dummy variables for the years under study. Table 1 details the definitions of the variables.

**Table 1:** Variable definition.

Variable		Definition
Dependent Variable	EI Pollution Control	Takes the value of 1 if an enterprise has pollution control investment, and 0 otherwise.
Independent Variable	GWCEO	Takes the value of 1 if a CEO has government work experience, and 0 otherwise.
Moderating Variables	ES Perception	The CEO’s economic status perception is coded as 1 to 10, with higher values indicating lower status perception. To handle reverse scoring, values above 5 are coded as 0, representing low economic status perception, while values below 5 are coded as 1, representing high economic status perception.
	SS Perception	The CEO’s social status perception is coded as 1 to 10, with higher values indicating lower status perception. To handle reverse scoring, values above 5 are coded as 0, representing low social status perception, while values below 5 are coded as 1, representing high social status perception.
	PS Perception	The CEO’s political status perception is coded as 1 to 10, with higher values indicating lower status perception. To handle reverse scoring, values above 5 are coded as 0, representing low political status perception, while values below 5 are coded as 1, representing high political status perception.
Control Variables	CEO Sex	CEO sex takes the value of 1 if the CEO is female and 0 otherwise.
	CEO Age	CEO age equals the year of the questionnaire minus the CEO’s birth year.
	CEO Political Connection	Takes the value of 1 if the CEO is a representative of the National People’s Congress or the Chinese People’s Political Consultative Conference, and 0 otherwise.
	Enterprise Age	The length of enterprise establishment equals the year of the questionnaire minus the year when the enterprise was registered as a private enterprise.
	Enterprise Industry	Enterprise industry takes a value of 1 if the industry experiences heavy pollution and 0 otherwise.
	Provincial GDP	The logarithm of provincial GDP in the previous year.
	Year	The year of the sample.
	Area	Takes the value of 1 if the area is in the eastern region, and 0 otherwise.

Notes: According to the guidelines for environmental information disclosure of listed enterprises (Exposure Draft) in 2010, combined with the characteristics of private enterprises, the mining, manufacturing, electricity, gas and water, construction, and transportation industries are heavy-pollution industries, while the rest are light-pollution industries.

http://doi.org/10.1371/journal.pone.0317903.t001

## 4. Results and analysis

Table 2 presents descriptive statistics and Pearson’s correlations. In our sample, the mean enterprise investment in pollution control is 0.355, indicating that enterprise investment in pollution control is generally low. The average value of CEOs with government work experience is 0.154, indicating that most CEOs of private Chinese enterprises in our sample have no government work experience. The variance inflation factor (VIF) values are all less than 3, and the mean VIF is 1.47, indicating multicollinearity between these variables is not a serious problem.

**Table 2:** Descriptive statistics and Pearson correlation coefficients.

	Mean	SD	1	2	3	4	5	6	7	8	9	10	11	12
1. EI Pollution Control	0.355	0.479	1											
2. GWCEO	0.154	0.361	0.023***	1										
3. ES Perception	0.589	0.492	0.147***	0.036***	1									
4. SS Perception	0.587	0.492	0.146***	0.036***	0.714***	1								
5. PS Perception	0.483	0.500	0.145***	0.046***	0.552***	0.670***	1							
6. CEO Sex	0.854	0.354	0.092***	0.010	0.073***	0.068***	0.074***	1						
7. CEO Age	46.037	8.392	0.104***	0.100***	0.104***	0.113***	0.140***	0.098***	1					
8. CEO Political Connection	0.663	0.473	0.136***	0.056***	0.131***	0.162***	0.231***	0.068***	0.104***	1				
9. Enterprise Age	9.472	5.427	0.150***	0.038***	0.152***	0.156***	0.178***	0.075***	0.345***	0.124***	1			
10. Enterprise industry	0.524	0.499	0.242***	-0.021**	0.109***	0.104***	0.104***	0.130***	0.154***	0.103***	0.149***	1		
11. Provincial GDP	28.144	0.832	0.094***	-0.040***	0.100***	0.081***	0.064***	0.052***	0.049***	-0.162***	0.154***	0.124***	1	
12. Area	0.577	0.494	0.008	-0.017**	0.075***	0.056***	0.033***	0.027***	0.016*	-0.010	0.127***	0.096***	0.525***	1

Note

*, **, *** indicate statistical significance at the 10%, 5%, and 1% level, respectively.

http://doi.org/10.1371/journal.pone.0317903.t002

Table 3 presents the analyses of the hypotheses. Model 1 contains only the control variables. Model 2 adds independent variables; Models 3 to 5 include status perception variables and the CEO and status perception interaction items. Hypothesis 1 posits that a CEO with government work experience positively correlates with enterprise investment in pollution control. Model 2 shows that a CEO with government work experience significantly impacts enterprise investment in pollution control (β = 0.106, p = 0.047 < 0.05). Therefore, H1 is supported.

**Table 3:** Regression results for CEOs with government work experience and enterprise investment in pollution control (Logit).

Variables	EI Pollution Control				
	Model 1	Model 2	Model 3	Model 4	Model 5
GWCEO		0.106**	0.110**	0.114**	0.116**
		(-0.054)	(-0.054)	(-0.054)	(-0.054)
GWCEO*ES Perception			-0.234**		
			(-0.112)		
GWCEO*SS Perception				-0.253**	
				(-0.112)	
GWCEO*PS Perception					-0.241**
					(-0.106)
ES Perception			0.419***		
			(-0.042)		
SS Perception				0.399***	
				(-0.042)	
PS Perception					0.328***
					(-0.041)
CEO Sex	0.331***	0.331***	0.309***	0.312***	0.316***
	(-0.060)	(-0.060)	(-0.060)	(-0.060)	(-0.060)
CEO Age	0.006**	0.005**	0.005*	0.004*	0.004
	(-0.003)	(-0.003)	(-0.003)	(-0.003)	(-0.003)
CEO Political Connection	0.754***	0.748***	0.683***	0.666***	0.648***
	(-0.053)	(-0.053)	(-0.053)	(-0.054)	(-0.054)
Enterprise Age	0.032***	0.032***	0.029***	0.029***	0.029***
	(-0.004)	(-0.004)	(-0.004)	(-0.004)	(-0.004)
Enterprise industry	0.928***	0.931***	0.916***	0.919***	0.922***
	(-0.041)	(-0.041)	(-0.041)	(-0.041)	(-0.041)
Provincial GDP	0.261***	0.263***	0.250***	0.254***	0.254***
	(-0.035)	(-0.035)	(-0.035)	(-0.034)	(-0.034)
Constant	-9.828***	-9.888***	-9.642***	-9.713***	-9.638***
	(-0.947)	(-0.948)	(-0.947)	(-0.945)	(-0.946)
Year	Yes	Yes	Yes	Yes	Yes
Area	Yes	Yes	Yes	Yes	Yes
Observations	12684	12684	12684	12684	12684
Pseudo R^2^	0.079	0.079	0.085	0.085	0.083

Note: Model 2 tests the relationship between CEOs with government work experience and enterprise investment in pollution control. Models 3 to 5 test the moderating effect of CEOs’ status perception.

*, **, *** indicate statistical significance at the 10%, 5%, and 1% level, respectively.

http://doi.org/10.1371/journal.pone.0317903.t003

Models 3 to 5 present the moderating role of CEO status perception. The regression results in column (3) of Table 3 show that CEOs’ economic status perception significantly and negatively moderates the relationship between CEOs with government work experience and enterprise investment in pollution control. The coefficient of the interaction term (GWCEO*ES Perception) is -0.234, passing the significance test at the 5% level (p = 0.037 < 0.05); thus, H2a is supported. This shows that low economic status perception weakens the positive relationship between CEOs with government work experience and enterprise investment in pollution control.

The regression results in column (4) of Table 3 show that CEOs’ social status perception significantly negatively moderates the relationship between CEOs with government work experience and enterprise investment in pollution control. The coefficient of the interaction term (GWCEO*SS Perception) is -0.253, passing the significance test at the 5% level (p = 0.024 < 0.05); thus, H2b is supported. This indicates that low social status perception weakens the government imprinting effect; that is, the positive impact of CEOs with government work experience on enterprise investment in pollution control is diminished by low social status perception.

The regression results in column (5) of Table 3 show that the CEO’s political status perception has a significant negative moderating effect on the relationship between CEOs with government work experience and enterprise investment in pollution control. The coefficient of the interaction term (GWCEO*PS Perception) is -0.241, passing the significance test at the 5% level (p = 0.024 < 0.05); thus, H2c is supported. This shows that low political status perception weakens the positive relationship between CEOs with government work experience and enterprise investment in pollution control.

**Fig 1:**
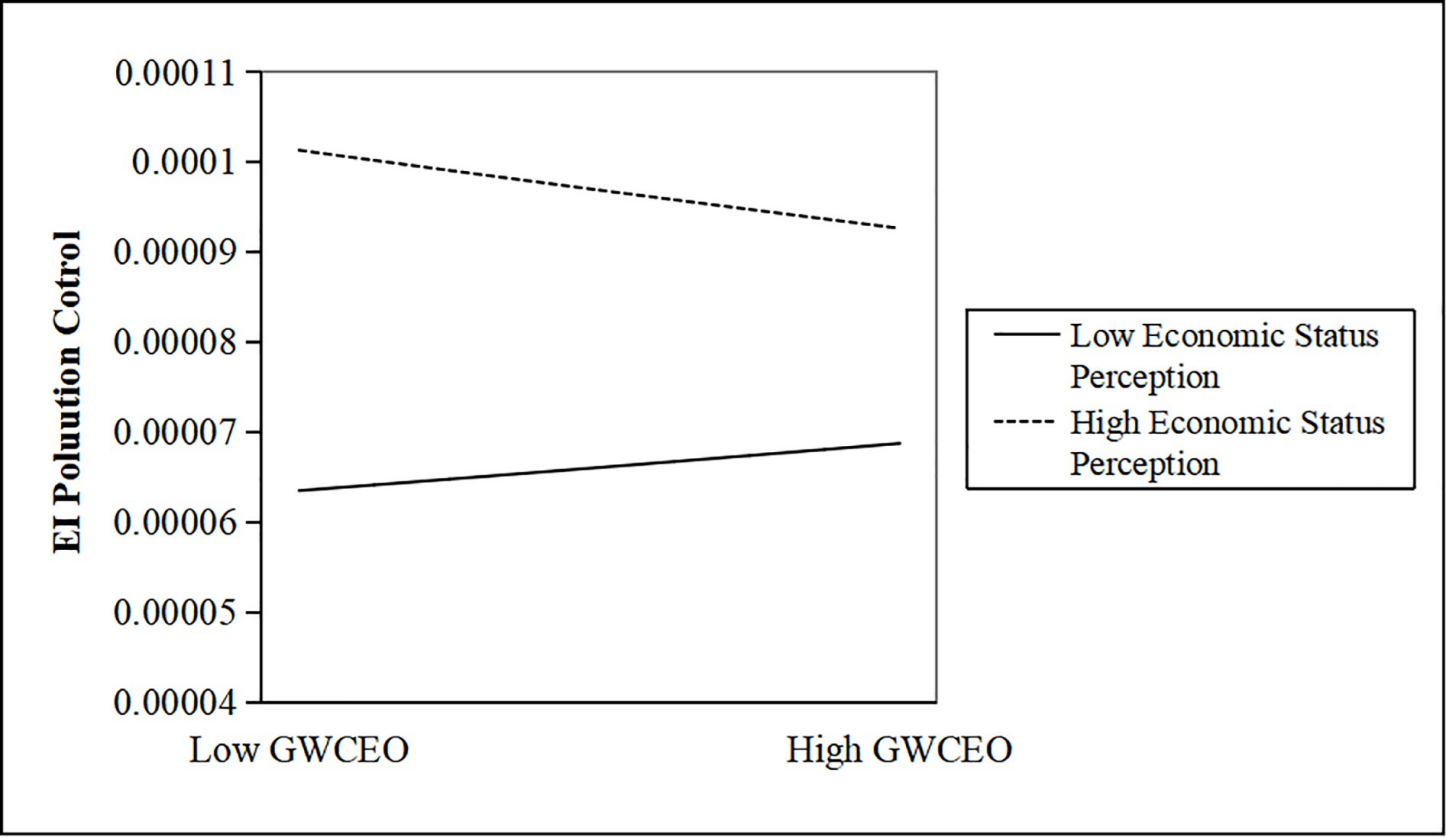
The moderating effect of a CEO’s economic status perception. http://doi.org/10.1371/journal.pone.0317903.g001.

**Fig 2:**
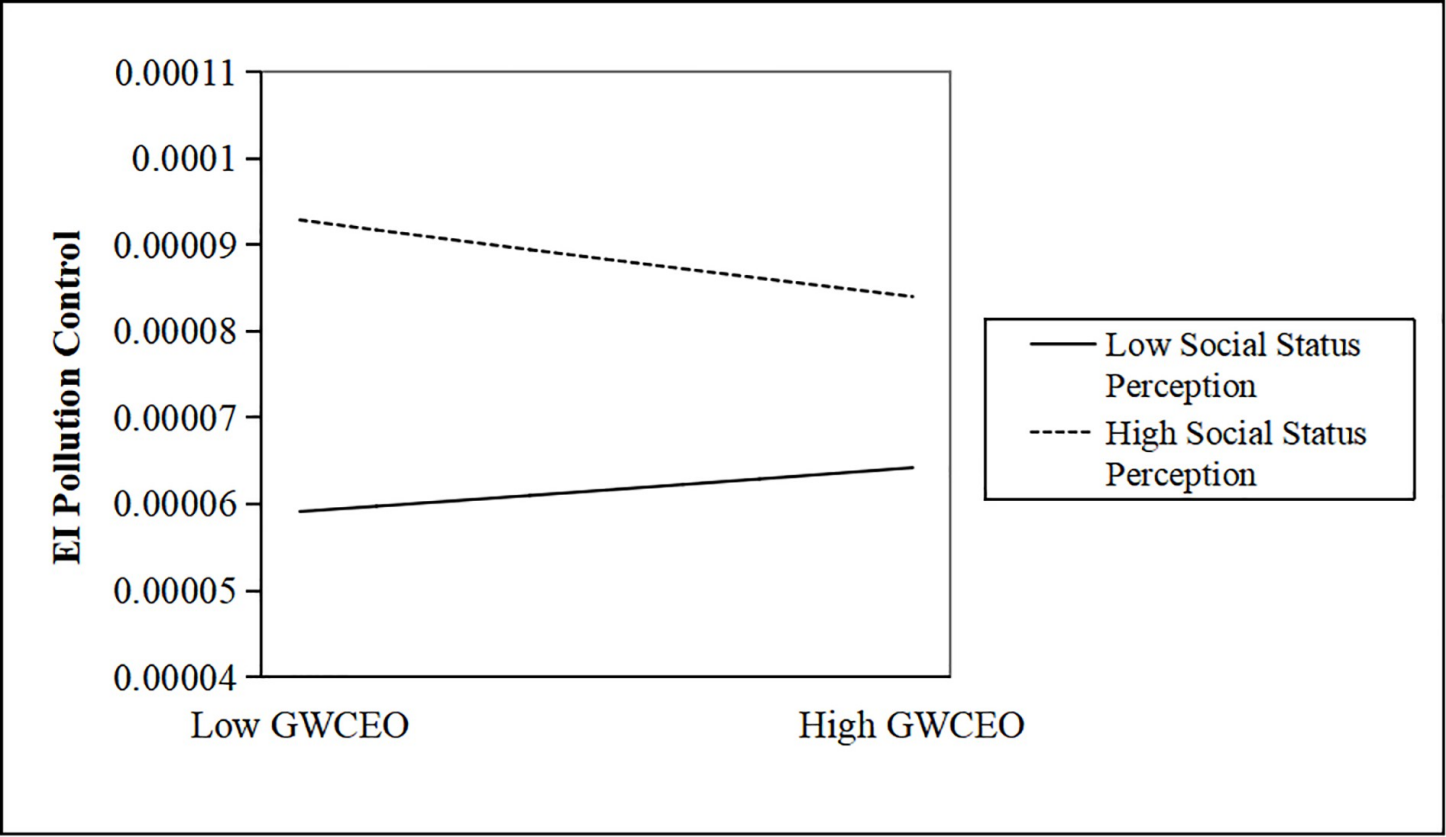
The moderating effect of a CEO’s social status perception. http://doi.org/10.1371/journal.pone.0317903.g002.

**Fig 3:**
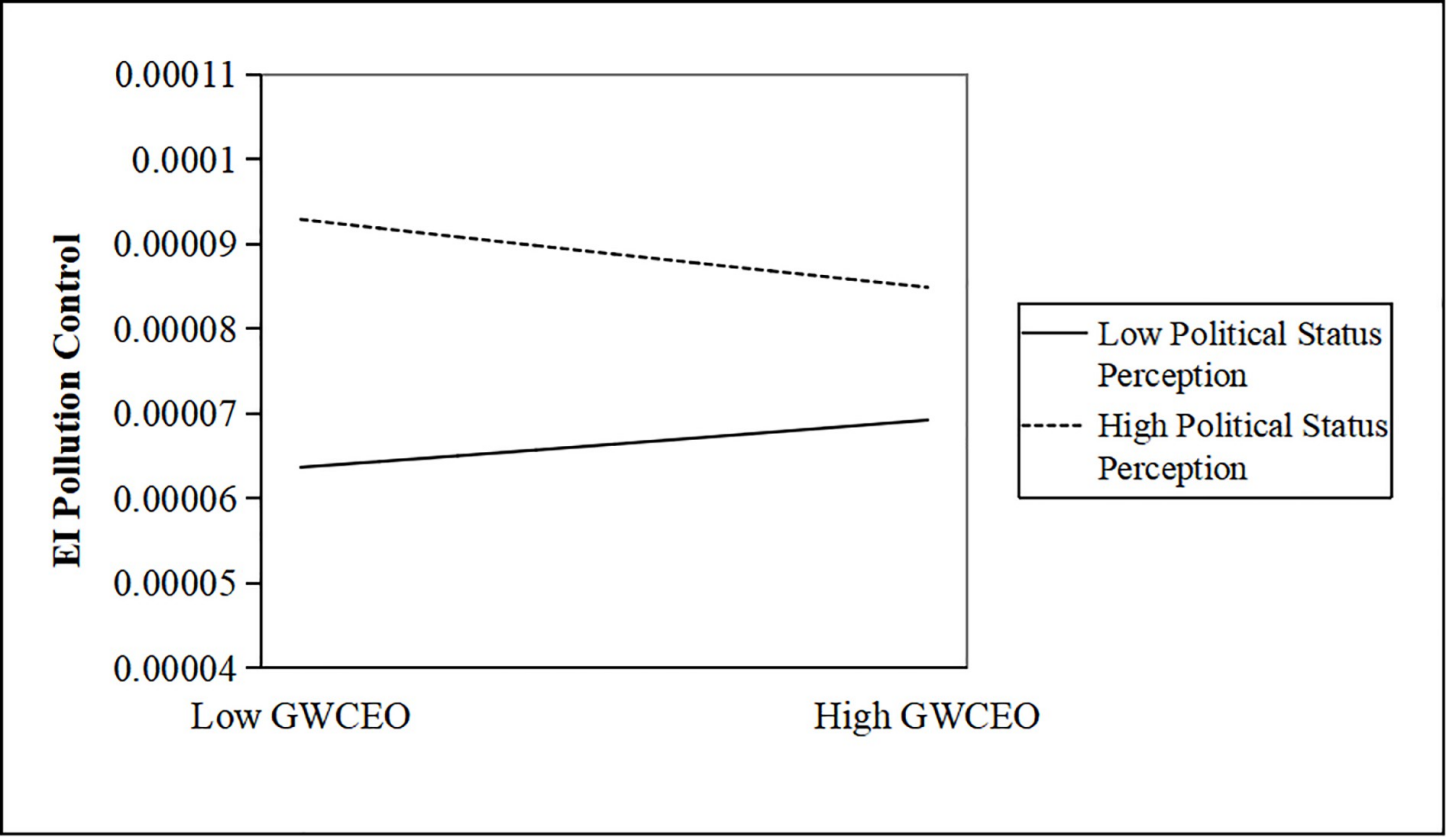
The moderating effect of a CEO’s political status perception. http://doi.org/10.1371/journal.pone.0317903.g003.

We further map the moderating effects. Figs 1–3 show that when CEOs have a low-status perception, as the number of CEOs with government work experience increases, enterprise investment in pollution control will be further weakened.

## 5. Robustness test and endogeneity concerns

Replacement regression method: probit regression. A probit regression is used to retest the main and moderating effects, and the results are shown in Table 4. CEOs with government work experience and enterprise investment in pollution control still show a significant positive correlation (β = 0.063, p = 0.054 < 0.1). The interaction coefficient between CEOs’ economic status perception and CEOs with government work experience is negative and significant (β = -0.142, p = 0.036 < 0.05). The interaction coefficient between CEOs’ social status perception and CEOs with government work experience is negative and significant (β = -0.156, p = 0.021 < 0.05). The interaction coefficient between CEOs’ political status perception and CEOs with government work experience is negative and significant (β = -0.145, p = 0.025 < 0.05). Except for the significance level of the main effect decreasing from 5% to 10%, the other results are consistent; thus, H1 to H2c are still supported.

**Table 4:** Probit regression results.

Variables	EI Pollution Control				
	Model 1	Model 2	Model 3	Model 4	Model 5
GWCEO		0.063*	0.064**	0.067**	0.067**
		(0.033)	(0.033)	(0.033)	(0.033)
GWCEO*ES Perception			-0.142**		
			(0.068)		
GWCEO*SS Perception				-0.156**	
				(0.068)	
GWCEO*PS Perception					-0.145**
					(0.065)
ES Perception			0.254***		
			(0.025)		
SS Perception				0.242***	
				(0.025)	
PS Perception					0.201***
					(0.025)
CEO Sex	0.201***	0.201***	0.188***	0.188***	0.191***
	(0.035)	(0.035)	(0.035)	(0.035)	(0.035)
CEO Age	0.004**	0.003**	0.003*	0.003*	0.003
	(0.002)	(0.002)	(0.002)	(0.002)	(0.002)
CEO Political Connection	0.458***	0.455***	0.414***	0.405***	0.393***
	(0.032)	(0.032)	(0.032)	(0.032)	(0.033)
Enterprise Age	0.019***	0.019***	0.018***	0.018***	0.018***
	(0.002)	(0.002)	(0.002)	(0.002)	(0.002)
Enterprise industry	0.562***	0.564***	0.555***	0.556***	0.558***
	(0.025)	(0.025)	(0.025)	(0.025)	(0.025)
Provincial GDP	0.150***	0.151***	0.143***	0.145***	0.146***
	(0.020)	(0.020)	(0.020)	(0.020)	(0.020)
Constant	-5.743***	-5.778***	-5.623***	-5.664***	-5.628***
	(0.551)	(0.551)	(0.551)	(0.550)	(0.551)
Year	Yes	Yes	Yes	Yes	Yes
Area	Yes	Yes	Yes	Yes	Yes
Observations	12684	1268	12684	12684	12684
Pseudo R^2^	0.079	0.079	0.086	0.085	0.083

Note

*, **, *** indicate statistical significance at the 10%, 5%, and 1% level, respectively.

http://doi.org/10.1371/journal.pone.0317903.t004

Endogeneity problems caused by sample selection: Heckman two-stage model. There may be endogeneity (or self-selection) between CEOs with government work experience and enterprise investment in pollution control. CEOs with government work experience may be determined by some external factors of the enterprise and the CEOs’ characteristics rather than randomly. To make our conclusion more reliable, we use a Heckman two-stage model to control for the endogeneity of the samples. In the first step of the probit regression analysis, we build the following model:

$$Probit(CEO∙Edu)=\Phi^{-1}(CEO∙Edu)=\gamma_{0}+\gamma_{1}∙CEO∙Gender+\gamma_{2}∙CEO∙Age+\gamma_{3}∙CEO∙Political∙Connection+\gamma_{4}∙Year+\gamma_{5}∙Area+\theta$$ (5)

The inverse mills ratio (IMR) calculated in the first step is used as the control variable in the second step of regression to address the samples’ endogeneity. The second-stage regression results in Table 5 show that the IMR is insignificant, indicating no endogeneity problem caused by sample deviation. Furthermore, the correlation coefficients of the main and moderating effects are still significant at the 5% level. Thus, we assume that H1 to H2c can still be verified.

**Table 5:** Results of the Heckman two-stage procedure.

**Panel A: The first-step regression model employed to estimate inverse mills**							
	**CEO Sex**	**CEO Age**	**CEO Political Connection**	**Cons**	**Year**	**Area**	**Observations**
CEO Edu	-0.305*** (0.033)	-0.138*** (0.001)	0.351*** (0.031)	-0.017*** (0.092)	Yes	Yes	12684
**Panel B: The second-step regression after introducing inverse mills**							
**Variables**			**EI Pollution Control**				
			**Model 1**	**Model 2**	**Model 3**	**Model 4**	**Model 5**
GWCEO				0.107**	0.110**	0.114**	0.116**
				(0.054)	(0.054)	(0.054)	(0.054)
GWCEO*ES Perception					-0.234**		
					(0.112)		
GWCEO*SS Perception						-0.252**	
						(0.112)	
GWCEO*PS Perception							-0.240**
							(0.106)
ES Perception					0.420***		
					(0.042)		
SS Perception						0.399***	
						(0.042)	
PS Perception							0.327***
							(0.041)
CEO Sex			0.301***	0.301***	0.272***	0.282***	0.288***
			(0.069)	(0.069)	(0.069)	(0.069)	(0.069)
CEO Age			-0.008	-0.009	-0.013	-0.010	-0.009
			(0.017)	(0.017)	(0.017)	(0.017)	(0.017)
CEO Political Connection			1.081***	1.081***	1.082***	0.994**	0.946**
			(0.392)	(0.392)	(0.394)	(0.392)	(0.393)
Enterprise Age			0.032***	0.032***	0.029***	0.029***	0.029***
			(0.004)	(0.004)	(0.004)	(0.004)	(0.004)
Enterprise industry			0.928***	0.931***	0.915***	0.918***	0.921***
			(0.041)	(0.041)	(0.041)	(0.041)	(0.041)
Provincial GDP			0.261***	0.263***	0.251***	0.254***	0.255***
			(0.035)	(0.035)	(0.035)	(0.034)	(0.034)
IMR			1.758	1.789	2.143	1.763	1.605
			(2.092)	(2.094)	(2.106)	(2.091)	(2.099)
Constant			-11.286***	-11.373***	-11.420***	-11.177***	-10.970***
			(1.987)	(1.989)	(1.997)	(1.985)	(1.993)
Year			Yes	Yes	Yes	Yes	Yes
Area			Yes	Yes	Yes	Yes	Yes
Observations			12684	1268	12684	12684	12684
Pseudo R^2^			0.079	0.079	0.085	0.085	0.083

Note

*, **, *** indicate statistical significance at the 10%, 5%, and 1% level, respectively.

http://doi.org/10.1371/journal.pone.0317903.t005

## 6. Discussion

This study draws on imprinting theory and status perception to explore the impact of CEOs with government work experience on enterprise investment in pollution control. Furthermore, it examines the moderating role of status perception. Based on data from Chinese private enterprises from 2008 to 2014, this study reached the following conclusions:

First, CEOs with government work experience have been proven to promote enterprise investment in pollution control. Previous research indicates that CEOs’ professional experience plays a significant role in enterprise development [33, 34], and this study aligns with that view. Gao et al. [33] found that CEOs’ financial experience hinders enterprise innovation, while Abecassis-Moedas et al. [34] suggested that CEOs’ entrepreneurial experience can influence an enterprise’s growth strategy. Unlike these two studies, which approach from an economic perspective, this study investigates the impact of CEOs’ non-commercial experience on enterprise development from an environmental perspective. The results show that CEOs with government work experience impart characteristics of government imprinting to their cognition and decision-making, thereby promoting enterprise investment in pollution control.

Second, economic, social, and political status perception negatively moderates the positive relationship between CEOs with government work experience and enterprise investment in pollution control. Although some scholars have found a positive relationship between status perception and enterprise environmental governance [27, 67], recent evidence suggests that the impact of status perception on enterprise environmental governance may be ambiguous [26, 97]. One limitation of these studies is their undifferentiated approach to status perception. Consistent with Weber [24], this study divides status perception into three dimensions to investigate and thoroughly reveal its impact on enterprise environmental governance. Accordingly, this study focuses on the weakening effects of three types of status perception on CEOs’ government imprinting. The results indicate that economic status perception weakens government imprinting through cognition and structure, social status perception weakens government imprinting through cognition and cuture, and political status perception weakens government imprinting through resources and strategy.

### 6.1 Theoretical contributions

Our study offers three main contributions. First, it explores the impact of CEOs with government work experience on enterprise investment in pollution control. This enriches the research on non-commercial experience career imprints and expands the application of imprinting theory in environmental governance. Marquis [20] posits that career imprints emphasize that early career experiences not only shape individual characteristics and behaviors but also influence their decision-making patterns and values in the long term. This process is significant during critical periods of career development when individuals selectively absorb characteristics, behaviors, and cognitive frameworks that reflect their imprints [19, 38]. Currently, most studies on career imprints and enterprise development focus primarily on individuals’ commercial experiences, such as financial experience [33], digital technology experience [98], and marketing experience [2]. However, as more CEOs transition from government sectors to the enterprise realm, the impact of their non-commercial experiences on enterprise development becomes more apparent. The knowledge, skills, and values accumulated during their government tenure influence their decision-making approaches and shape their perceptions and responsibilities regarding enterprise social responsibility [42, 99]. Nevertheless, research on non-commercial experience career imprints remains insufficient. Most literature must adequately explore how government work experience shapes CEOs’ decision-making behaviors and how these behaviors affect enterprise investment in pollution control [4, 11]. Therefore, this study defines the decision-making behaviors formed by individual government work experience as “government imprinting,” focusing on how government imprinting influences CEOs’ decisions regarding enterprise investment in pollution control. This perspective expands the research on non-commercial experience career imprints and provides new opportunities for the further development and application of imprinting theory in environmental governance.

Second, our study contributes significantly to the literature on status perception by delineating its dimensions and exploring how they influence the relationship between CEOs with government work experience and enterprise investment in pollution control. Previous research has focused on the impact of status perception on enterprise development. For instance, Sheng et al. [91] examine the effect of CEOs’ subjective status perception on international behaviors, while Li et al. [67] investigate the influence of CEOs’ self-perceived status on enterprise social responsibility. However, the lack of dimensional differentiation has limited further in-depth research in status perception [30], resulting in insufficient theoretical guidance for enterprises [29]. Existing research has indicated that various dimensions of status perception can affect CEOs’ decision-making styles. For instance, CEOs with high economic status perception are more likely to focus on short-term financial performance [25], while those with high social status perception emphasize enterprise social reputation [26]. Therefore, this study categorizes status perception into three dimensions: economic, social, and political, and further explores the moderating effects of each dimension on the relationship between CEOs with government work experience and enterprise investment in pollution control. This study enriches the conceptual connotation and practical application of status perception, provides essential references for enterprises to flexibly utilize status perception in different contexts, and lays the foundation for subsequent research on the complex mechanisms of status perception in enterprise development.

Third, this study explores the impact of CEOs with government work experience on enterprise investment in pollution control from a micro-level perspective, making a significant contribution to the literature on sustainable development. Previous research has primarily focused on macro- and meso-level factors [100, 101], such as environmental legislation [88], environmental regulation [102], digital transformation [103], enterprise social responsibility [104], product innovation [105], and organizational culture. However, as leaders of enterprises, CEOs’ personal experiences, values, and decision-making styles significantly influence the decision-making and execution effectiveness of enterprise environmental investment, thereby influencing sustainable development [1, 3, 52]. Currently, research on the impact of CEOs on enterprise environmental governance primarily concentrates on two aspects: one is the innate traits of CEOs, such as gender [5], age [6], and overconfidence [7]; and the other is their acquired experiences, including marketing [2], finance, and overseas experiences [106]. Environmental behaviors driven by these acquired experiences are typically oriented toward serving enterprise interests, manifested as externally motivated actions. Conversely, CEOs with government work experience can foster a sense of social responsibility and mission for environmental protection [17, 18], both cognitively and structurally. This intrinsic sense of responsibility is more likely to influence long-term enterprise environmental governance behaviors, thus promoting sustainable development [4, 50], which warrants deeper investigation. Additionally, this study responds to the call by Mahran and Elamer [4] regarding how CEO experience affects environmental sustainability practices and outcomes. The recent controversies surrounding Japan’s nuclear wastewater discharge have sparked global discussions, prompting academia and practice to emphasize the significance of pollution control investment in sustainable development. Therefore, this study focuses on the impact of CEOs with government work experience on enterprise investment in pollution control at the micro level. Such experiences instill a spontaneous sense of responsibility in CEOs, motivating them to strive for sustainable development and providing new perspectives and insights for research in this area.

### 6.2 Practical implications

First, enterprises can hire CEOs with previous government work experience to help them respond to government requirements for environmental protection. Our findings indicate that CEOs with government work experience can promote enterprise investment in pollution control. These CEOs, who have previously worked in government departments, can interpret government environmental policies and dynamics more thoroughly and possess richer experience in environmental pollution control [3, 49]. Therefore, enterprises should explicitly recruit CEOs with government work experience and encourage them to engage in exchanges with diverse partners, actively participate in internal and external strategic development training, and fully leverage their advantages in past government work experience while addressing any shortcomings in enterprise management experience.

Second, enterprises must understand how the CEO’s status perception influences decision-making, primarily regarding environmental investments. Research indicates that economic, social, and political status perceptions negatively moderate the relationship between CEOs with government work experience and enterprise investment in pollution control. Therefore, enterprises should conduct regular surveys and foster communication to gain deeper insights into CEOs’ status perception preferences and develop strategies to encourage more proactive environmental investment decisions [107]. For instance, concerning economic status perception, enterprises could implement economic incentive policies linked to the performance of environmental investments, such as bonuses and other financial rewards, to motivate CEOs to prioritize environmental projects [4]. These incentive measures can help alleviate the short-term focus of high-status CEOs in resource allocation, guiding them toward more long-term investments in pollution control.

Third, enterprises can establish a continuous feedback mechanism to understand the CEO’s understanding and attitude toward environmental policies and dynamics and adjust the enterprise’s pollution control investment strategy based on the feedback results. Research findings indicate that all three types of CEO status perception can influence enterprise investment in pollution control. Therefore, enterprises can establish regular CEO environmental policy seminars or working groups, allowing CEOs to discuss environmental policies and dynamics with diverse partners such as environmental experts and government officials. This regular discussion mechanism can promote CEOs’ attention and reflection on environmental issues, identify problems, and propose solutions promptly, driving continuous progress in the enterprise’s environmental efforts.

### 6.3 Limitations and future research

First, future research can expand the study of imprinting theory in terms of research subjects and contexts. This study primarily focuses on how the decision-making patterns of CEOs, as part of the management, create government imprinting effects on enterprise investment in pollution control. However, an enterprise is composed not only of its management but also of its employees. As essential executors, employees are decisive in implementing enterprise investment in pollution control [108]. Therefore, future scholars should pay more attention to studying the imprinting effects generated by the group decision-making patterns of enterprise employees on pollution control investments [19]. Additionally, this study does not consider the impact of the macro-external environment on enterprise investment in pollution control. With the advent of new-generation information technology, the iteration of various production factors within enterprises is accelerating. How the continuous influence of CEO imprinting affects pollution control investment decisions will unfold in the face of dynamic changes in digital technology is a new issue worth paying attention to in the digital era [103, 109].

Second, future research can employ diverse data and various research methods to delve into the dynamic process of imprinting effects. This study uses mixed cross-sectional data for empirical research, focusing primarily on the moderating role of CEOs’ status perception on government imprinting. Future research could utilize dynamic panel data to capture the long-term impact paths of CEO government imprinting on enterprise investment in pollution control at different time points [9]. Additionally, future studies could consider adopting qualitative research methods, such as case studies [110], to investigate how CEO government imprinting affects enterprise investment in pollution control decisions and outcomes. Applying these diverse data and research methods will help to more comprehensively understand the application of imprinting theory in enterprise environmental decision-making, thereby providing theoretical and practical guidance for enterprises to make effective decisions in a dynamic environment.
